# Low Density Wood-Based Particleboards Bonded with Foamable Sour Cassava Starch: Preliminary Studies

**DOI:** 10.3390/polym8100354

**Published:** 2016-10-08

**Authors:** Sandra Monteiro, Jorge Martins, Fernão D. Magalhães, Luísa Carvalho

**Affiliations:** 1 LEPABE—Faculdade de Engenharia, Universidade do Porto, rua Dr. Roberto Frias, 4200-465 Porto, Portugal; sandram@fe.up.pt (S.M.); jmmartins@estgv.ipv.pt (J.M.); fdmagalh@fe.up.pt (F.D.M.); 2 DEMAD—Department of Wood Engineering, Polytechnic Institute of Viseu, Campus Politécnico de Repeses, 3504-510 Viseu, Portugal

**Keywords:** lightweight, particleboards, sour cassava starch, foam

## Abstract

This work investigates the feasibility of producing low density particleboards using an adhesive system based on sour cassava starch, taking advantage of its adhesive and self-expansion properties. Relevant properties of the produced particleboards were evaluated according to European Standards including: density, internal bond, moisture content and thickness swelling. Low density particleboards were produced with densities between 207 kg/m^3^ and 407 kg/m^3^. The best performance corresponded to particleboard with a density of 318 kg/m^3^, an internal bond strength of 0.67 N/mm^2^, and a thickness swelling of 8.7%. These values meet the standard requirements of general purpose lightweight boards for use in dry conditions. Heat post-treatment (24 h at 80 °C) led to lower internal bond strength, due to retrogradation (recrystallization of amylose and amylopectin chains upon cooling) causing higher rigidity of the starch binder. However, it showed to have a significant effect on decreasing the thickness swelling.

## 1. Introduction

According to Mohanty, “sustainability, industrial ecology, eco-efficiency, and green chemistry are guiding the development of the next generation of materials, products, and processes.” There is a vital need for development of innovative bio-based products and technologies that are not dependent on fossil fuel [1]. Biomaterials, obtained partially or entirely from biomass sources, play a relevant role in this context [2]. European standard EN 309 defines particleboard as a “panel material manufactured under pressure and heat from particles of wood (wood flakes, chips, shavings, saw-dust and similar) and/or other lignocellulosic material in particle form (flax shives, hemp shives, bagasse fragments and similar), with the addition of an adhesive” [3]. Lately, special attention has been given to lightweight particleboards, in an effort to facilitate handling and transportation. Furthermore, there is a current trend in furniture design to use high-thickness components, further increasing the demand for materials with reduced weight. Panels with a density below 600 kg/m^3^ are defined as lightweight particleboard (CEN/TS 16368) [4]. One way to decrease the weight of panels is by incorporating low density particles in the core layer [5]. However, this comes along with decreases in mechanical performance (flexural strength, internal bond strength, and resistance to axial withdrawal of screws), in the quality of the finish (coating, edge lamination), and in machinability.

Synthetic formaldehyde-based resins are currently used to produce wood-based particleboards, due to their low cost and good binder performance. Natural-based alternatives have been reported, such as adhesives based on tannins, lignin and their combinations [6,7,8], soy protein [9,10,11,12] and starch [13,14]. Unlike tannins, the other natural adhesives must be combined with a synthetic compound, such as phenol-formaldehyde resin or PMDI (polymeric isocyanate), in order to satisfy the requirements of relevant standards [15].

**Figure 1:**
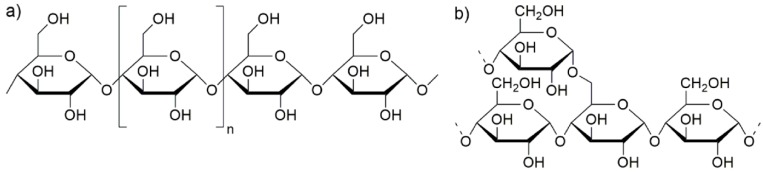
Molecular structure of (**a**) amylose and (**b**) amylopectin.

Starch is one of the most abundant natural polymers and is relatively inexpensive. Cereal grains (such as corn and wheat), tubers (such as potato), and roots (such as cassava) are some of the commercial sources of starch for industrial exploitation. Starch consists of two major molecular components, amylose and amylopectin (Figure 1), which can be differentiated by their chemical structure. The linear α-(1→4) linked glucan is called amylase while an α-(1→4) linked glucan with 4.2%–5.9% α-(1→6) branch linkages is amylopectin.

Starch has excellent affinity towards polar materials such as cellulose, because of the many hydroxyl groups, forming strong adhesive bonds [16]. It is extensively used in the form of binders, sizing materials, glues, and pastes [17]. Its current use in particleboard production involves a combination with synthetic adhesives, such as isocyanates and urea-formaldehyde resins [18].

Cassava sour starch is a traditional food ingredient made by fermentation and sun drying of native cassava starch. It shows good expansion upon heating, which makes it widely used in baking. Expansion occurs after starch gelatinization, as a consequence of water vaporization combined with appropriate paste fluidity, swelling and solubilization. Presence of carboxylate functional groups, formed during fermentation/sun drying, has been correlated with expansion performance [19]. Some authors studied the production of biodegradable foam trays based on cassava starch blended with chitosan and Kraft fiber [20] or with sunflower protein and cellulose fiber [21]. Cassava starch, in combination with a small percentage of urea-formaldehyde resin, has been used as a binder in wood-based particleboards with densities of 650 and 750 kg/m^3^ [22].

The world’s largest producers of cassava are Africa with 57% and Asia with 31%. Latin America and the Caribbean provide 12% of the world’s supply [23]. Approximately 60% of the production is used for human consumption, about 33% for animal feed and 7% for different industries such as textile, paper, food and adhesives [24]. The uses of cassava vary depending on the continent. While in Asia it is used mostly in industry, in Africa its main purpose is human consumption. Even though the cassava world trade grows about 36% per year, its price has remained stable and can actually be considered low when compared to other crops. It can be argued that cassava can be used on a large scale as a raw material for different products without compromising its consumption as food or the local ecosystems. An important factor that contributes to this observation is that cassava can be produced in low fertility soils and where the drainage is insufficient [25], unlike corn or potato. This allows taking advantage of currently deteriorated and unused land for cassava production [23]. In addition, cassava can be stored for long periods of time [26]. According to the report of the United Nations, in some African countries there is a political interest in making cassava a driving force of local economic growth [27]. Agriculture must be viewed not only as a means of subsistence but also as a foundation for the integrated and sustainable industrial production of food, energy and biosourced materials [23,28]. The present work studies the feasibility of producing low density particleboards using sour cassava starch as the main binder, taking advantage of its adhesive and expansion properties. The resulting 100% natural products were characterized in terms of their physico-mechanical properties.

## 2. Materials and Methods

### 2.1. Materials

Native and sour cassava starch was purchased at a local supermarket in Porto, Portugal. Chitosan (molecular weight around 300 kDa, degree of deacetylation > 85%) was supplied by Golden-Shell Pharmaceutical Co. Ltd (Zhejiang, China). *Populus* L. fibers, commonly used in medium density fiber panels (MDF) production, was provided by Valbopan-Fibras de Madeira S.A (Nazaré, Portugal). Glycerol (≥95%) was supplied by Sigma-Aldrich (Steinheim, Germany). Wood particles with a moisture content of 4% used for the production of particleboards were supplied by Sonae Indústria PCDM (Oliveira do Hospital, Portugal).

### 2.2. Determination of Amylose Content

The amylose content determination was performed by Eurofins (Madrid, Spain). Sample was first defatted with dichloromethane/methanol and 75% of propanol, and then the starch was solubilized in 2 M sodium hydroxide solution. The amylose content was determined by potentiometric titration with in-situ formed I_2_ (from iodate). This procedure is based on the method described by Banks et al. [29].

### 2.3. Scanning Electron Microscopy

The interior morphology of particleboards was observed by scanning electron microscopy, using a high resolution (Schottky) Environmental Scanning Electron Microscope with X-ray Microanalysis and Electron Backscattered Diffraction analysis: Quanta 400 FEG ESEM/EDAX Genesis X4M (FEI, Hillsboro, OR, USA). Samples were coated with a Au/Pd thin film, by sputtering, using the SPI Module Sputter Coater equipment. The analysis were performed at CEMUP (Centro de Materiais da Universidade do Porto, Porto, Portugal).

### 2.4. Fourier Transform Infrared (FTIR) Spectroscopy

FTIR spectra of the native and sour cassava starch were recorder with a Vertex 70 spectrometer (Bruker, Karlsruhe, Germany). The absorbance spectra were computed between 4000 and 500 cm^−1^ at 4 cm^−1^ resolution. Symmetrical interferograms on 66 scans were co-added for each spectrum.

### 2.5. Preparation of Binder System

The formulation of the adhesive system is shown in Table 1. Sour cassava starch and distilled water were mixed first. Chitosan solution, at 5 wt % concentration, was prepared by adding chitosan to propionic acid solution (6 wt %), and then mixing for 3 h at 60 °C. This solution was then added to the binder adhesive in the proportion shown in Table 1. Finally, *Populus* fibers and glycerol were added and stirring was maintained for 5 min. The adhesive system is a brownish viscous liquid with a solid content of 38%.

### 2.6. Foamability of Sour and Native Cassava Starch

Two formulations were prepared with each starch, as described in Section 2.5. The same amount of mixture (20 g) was placed in a glass beaker, and heated in an oven at 190 °C during 40 min. The volume of expansion was measured.

### 2.7. Particleboard Production

Wood particles were manually blended with the adhesive. The adhesive/wood ratio was 1:1, based on weight of solid adhesive content and oven dry wood. Single layer mats were hand formed in square aluminum foil deformable containers (Lusoforma, Mem Martins, Portugal), with initial dimensions of 220 × 220 × 80 mm^3^. A computer-controlled laboratory scale hot-press (homemade at our laboratory), equipped with a linear variable displacement transducer (LVDT), a pressure transducer and thermocouples, was used for producing the panels. The platen temperature was set to 190 °C. The adhesive/wood mixture was placed on the bottom platen, and the top platen was positioned at 25 mm height, during 30 min. Afterwards the top platen was slowly lifted until 28 mm thickness, over a 6 min period, and the final position maintained for 5 min.

About 12 h after production, part of the particleboards were subjected to heat post-treatment (HPT) in an oven, at 80 °C for 24 h. The remaining boards were not subjected to any kind of post-treatment. All boards were conditioned at 20 °C and a relative humidity of 65% until testing. The mass of wood/binder mixture used to produce the panels with different densities was between 276 g and 725 g.

### 2.8. Particleboard Characterization

The boards were tested according to the European standards for density (D) (EN 323), internal bond strength (IB) (EN 319), moisture content (MC) (EN 322) and thickness swelling (TS) (EN 317). In order to evaluate the variability of the measurements, 5 particleboards were produced from the same binder formulation and their properties measured. The coefficient of variation (ratio between standard variation and average value of measurement) obtained for each property were: density—4.0%, internal bond strength—2.7%, thickness swelling—10.7%, moisture content—7.5%.

## 3. Results and Discussion

### 3.1. Foamability of Sour Cassava Starch

**Figure 2:**
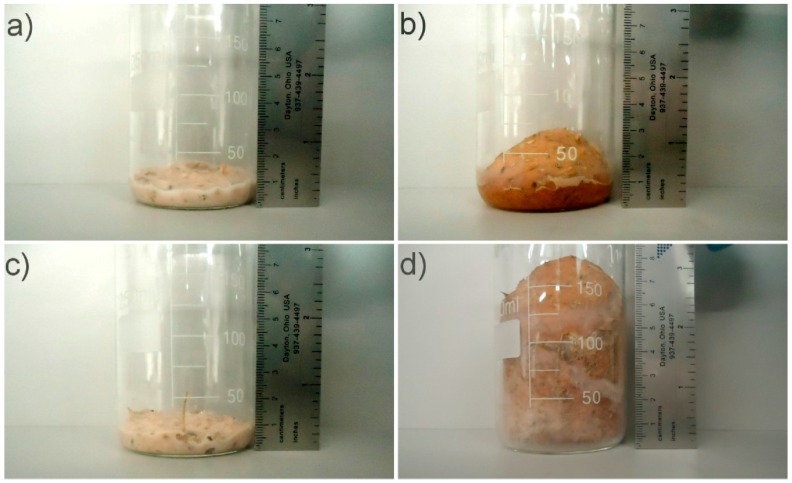
Expansion behavior of different types of cassava starches. (**a**,**b**) Native cassava starch before and after heating, respectively; (**c**,**d**) sour cassava starch before and after heating, respectively.

The ability of native and sour cassava starches to expand during baking was compared. The formulation containing sour cassava starch increased about 700% in volume (Figure 2), while the one containing native starch increased about 200%. Water vaporization combined with good swellability and paste fluidity of sour cassava starch led to the formation of a foam with good a expansion ability upon heating, without the need for the addition of expansion agents [19].

### 3.2. FTIR Spectroscopy Analysis

**Figure 3:**
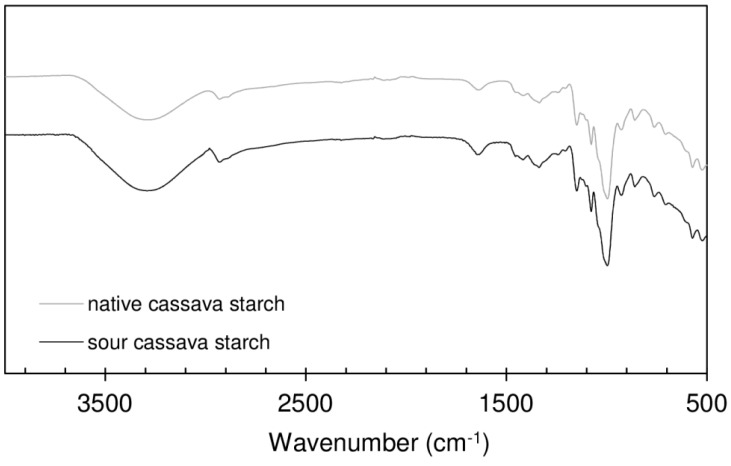
FTIR spectra of native and sour cassava starch in the (4000–500 cm^−1^) spectral region.

Figure 3 represents the FTIR spectra of native and sour cassava starches. The two spectra are very similar. The absorption peaks at 3291.2 cm^−1^ and 2930.7 cm^−1^ can be attributed to O–H and C–H bond stretching, respectively [30]. The absorption at 1645.0 cm^−1^ was assigned as C–O–C stretching and the absorption peak at 1455.4 cm^−1^ was attributed to C–H bending.

The absorbances at 1149.4 cm^−1^ and 1077.2 cm^−1^ are both assigned as the coupling of C–O, C–C and O-H bond stretching, bending and asymmetric stretching of the C–O–C glycosidic bridge [31]. The absorbance peak at 997.2 cm^−1^ can be attributed to the C–O–H bending and H–C–H related modes. The peak at 860.1 cm^−1^ can be assigned to C–O–C symmetrical stretching and C-H deformation [32].

Sour starch was expected to show higher carbonyl and carboxyl content than native starch, as a result of oxidation during the fermentation/sun drying production process [19]. Such differences are, however, not visible, in particular in terms of the expected carbonyl band at 1735 cm^−1^. Similar results were obtained by Demiate et al. [33] and may be due to a low concentration of said groups not allowing detection.

The amylose content of native and sour cassava starches was determined by potentiometric titration, and a value of 18% (*w*/*w*) was obtained for both.

### 3.3. Physico-Mechanical Properties of Particleboards

**Figure 4:**
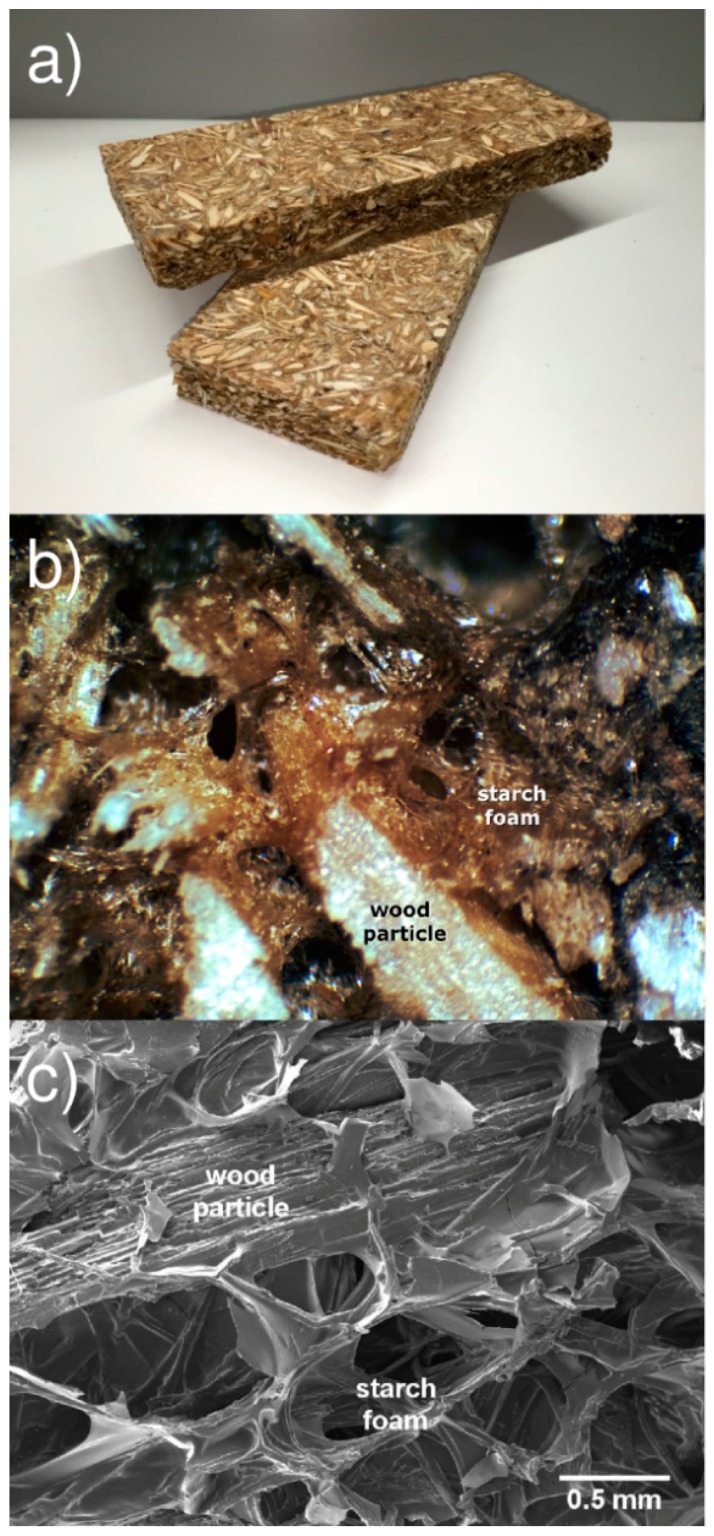
Particleboards produced with sour cassava starch–based binder. (**a**) Outside appearance; (**b**) close-up detail; (**c**) SEM image showing wood particles and surrounding foam, 100× magnification.

Figure 4 depicts the particleboards produced with sour cassava starch–based binder. The boards, shown in Figure 4a, appear rigid and strong when handled, and have no noticeable smell after cooling. The wood particles show good cohesion and cannot be easily detached. Figure 4b shows that the particles are surrounded by a heterogeneous foam-like material, created by starch expansion. The internal morphology of the panel is seen more clearly in the SEM image depicted in Figure 4c. The foam fills the voids between the particles and seems to be well adhered to the wood particle surfaces. Wood fibers, present in the adhesive formulation, are visible within the foam cell walls. The binder’s foaming ability causes separation of the wood particles, allowing for the panel’s low density, hopefully maintaining good cohesion and therefore guaranteeing acceptable mechanical properties. Chitosan and wood fiber were added to the sour cassava starch formulation in order to improve the foam properties, based on previous evidence found in the literature [20]. In addition to the reinforcing role of wood fiber, highly compatible with the starch matrix, the interaction between the amino groups of chitosan and the hydroxyl groups of starch may provide mechanical strengthening and decreased water solubility. The formulation has not yet been fully optimized for this application, the purpose of the present work being to demonstrate the feasibility and potential of low density particleboards produced using this type of binder.

**Figure 5:**
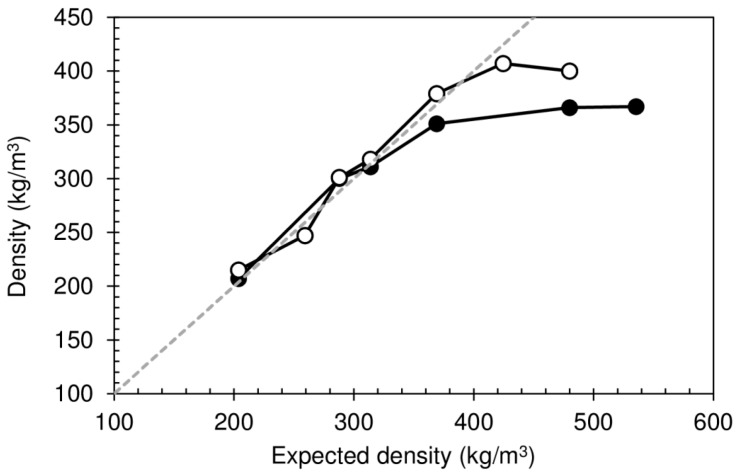
Density of dry particleboards bonded with sour cassava starch foam. White circles: without HPT; black circles: with HPT. Dashed line shows coincidence between measured and expected densities.

The particleboards were produced from different starting masses of wood/binder mixture, in order to identify an optimal combination of composite properties. The effect of the heat post-treatment (HPT of 80 °C for 24 h) after production was also evaluated. The results of the density of the final dry particleboards are shown in Figure 5, as a function of the expected density (ratio of the dry mixture mass to the volume corresponding to the thickness defined by the final press platen separation of 28 mm).

**Figure 6:**
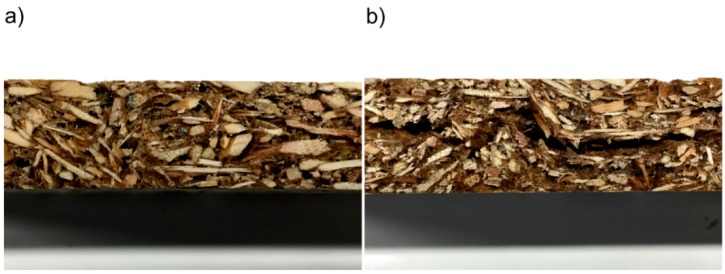
Detail of internal morphology of particleboards: (**a**) expected density of 313 kg/m^3^, showing uniform internal structure; (**b**) expected density of 550 kg/m^3^, showing internal delamination.

The minimum value of the density obtained was 215 kg/m^3^. Initially the densities of the particleboards increase with the increase of the total mass of mixture used, independent of being subjected or not to the heat post-treatment. The mixture expands until reaching the top press platen, which defines the maximum height of the particleboard. The measured values coincide with the expected ones: Greater mixture mass placed between the press platen implies higher density. However, for expected densities above about 350 kg/m^3^, the measured densities show a negative deviation. This is a consequence of the high amount of water vapor that is retained within the panel while it stays inside the press. After removal, this internal pressure results in a springback effect, causing the particleboard to expand, thus decreasing its density. For expected densities above around 550 kg/m^3^, this actually tends to cause internal bursting, since the cohesive strength of the binder, still warm and with high moisture content, is not sufficient to balance the internal stress caused by trapped vapor. Figure 6 shows close-up images of two boards, one produced with a low initial mass (a), showing uniform internal morphology, and another that has visibly delaminated due to the phenomena described above (b).

No significant difference was found in the equilibrium moisture content of the particleboards. This was in the range of 10.4% to 12.3% for non-heat-treated boards, and of 8.4% to 11.6% for heat-treated boards.

**Figure 7:**
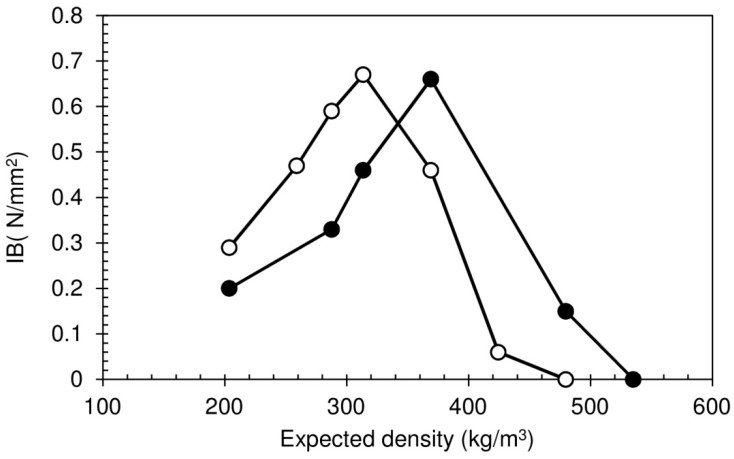
Internal bond of particleboards bonded with sour cassava starch foam. White circles: Without HPT; black circles: With HPT.

The internal bond strength (IB) provides an overall evaluation of the board’s mechanical integrity, indicating how well the core material is bonded together. The IB values of the particleboards produced are given in Figure 7. Particleboards not subjected to HPT show an initial increase in the IB, as expected from the increased density, as it promotes more effective internal cohesion. A maximum value of 0.67 N/mm^2^ was attained, corresponding to a density of 318 kg/m^3^. Note that according to European Technical Specification CEN/TS 16368, for lightweight particleboards type LP2 (the most demanding) with thicknesses between 20 and 25 mm, the minimum requirement for the IB is 0.30 N/mm^2^ [4].

For expected densities above 313 kg/m^3^, the IB decreases, due to the higher board density causing vapor entrapment and, as a consequence of the resulting internal stress, rupture of inter-particle bonds. For the highest expected densities, this ends up leading to full delamination, as discussed before, and a virtually null IB. Heat post-treatment has two distinct effects on the IB of the particleboards. For expected densities up to 313 kg/m^3^, it leads to a lower IB. Heat treatments are used for the physical modification of starch granules, inducing crystalline rearrangement and promoting intramolecular bonds [34].

The actual outcomes of the treatment are highly dependent on the starch’s botanical origin [35]. In the work presented here, HPT causes some degree of modification in the sour cassava starch that yields a weaker foam structure, probably due to increased brittleness. The heat treatment may be inducing retrogradation, i.e., recrystallization due to the rearrangement and intramolecular interactions during the expulsion of water. This is observed in the aging of thermoplastic starch, which is known to result in increased brittleness [36]. Interestingly, for the higher expected densities, the IB obtained for the heat-treated particleboards is actually higher than for the non-treated ones. The reasons for this strengthening are not yet clear, but may be related to the higher moisture content of these boards, shortly after production, resulting in a different physical rearrangement of starch during the heat treatment.

**Figure 8:**
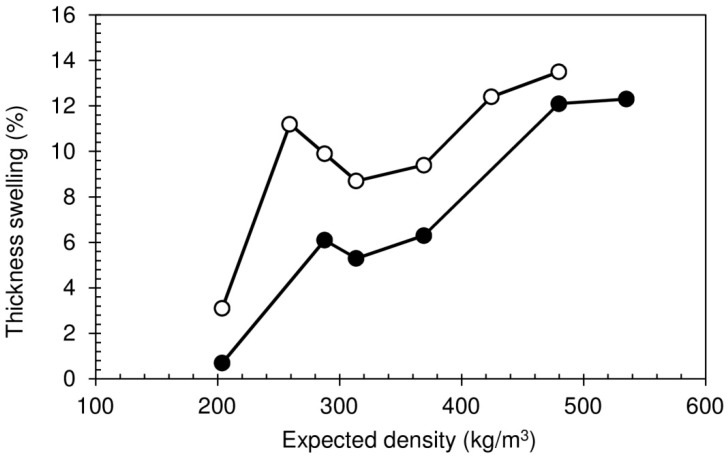
Thickness swelling of particleboards bonded with sour cassava starch foam. White circles: Without HPT; black circles: With HPT.

The thickness swelling (TS) results are shown in Figure 8. The TS tends to increase with the expected density, due to higher density causing higher water absorption. However, this effect is counterbalanced by the improvement in the internal bond strength, resulting in the dip in TS for expected densities between 300 and 350 kg/m^3^, where the IB shows a maximum value. Since the IB decreases for higher densities, thickness swelling increases again. It must be noted that the values obtained for the TS can be considered quite low. According to European Standard EN 312, for non-load-bearing boards for use in humid conditions with thicknesses between 20 and 25 mm, the maximum acceptable value for TS is 13% [37]. For the panel produced with a density of 318 kg/m^3^, which displayed the best IB result, the measured TS is 8.7%. It is important to note that these boards are intended for furniture use in dry conditions (standard value not specified).

The heat post-treatment consistently causes a significant decrease in TS. Once again, this may be related to the physical modifications that are known to occur in starches subject to heating (increased crystallinity, reduced hydration, increased intramolecular interaction), which translate into reduced swelling power [34].

## 4. Conclusions

Lightweight particleboards, bonded with an adhesive system, based on sour cassava starch with added chitosan and wood fibers, were produced with densities between 207 kg/m^3^ and 407 kg/m^3^. These low density values were obtained thanks to the good self-foaming property of the starch, which allows for the separation of wood particles and the creation of low density domains while maintaining good cohesion. Very good internal bond strength and thickness swelling values were obtained. The best overall performance corresponded to a panel with a density of 318 kg/m^3^, an IB of 0.67 N/mm^2^, and a TS of 8.7%. Heat post-treatment revealed to not be beneficial in terms of internal bond strength, but led to a decrease in the thickness swelling.

The use of this 100% natural adhesive system allows for producing low density particleboards with excellent performance. Optimization of the adhesive formulation and production conditions will be the subject of future studies.

## Figures and Tables

**Table 1:** Formulation of the adhesive system.

Component	Quantity (wt %)
Sour cassava starch	30.5
Distilled water	30.5
Chitosan solution	33.4
Populus fibers	2.8
Glycerol	2.8
